# Efficacy of Nutritional Interventions on Inflammatory Markers in Haemodialysis Patients: A Systematic Review and Limited Meta-Analysis

**DOI:** 10.3390/nu10040397

**Published:** 2018-03-23

**Authors:** Ban-Hock Khor, Sreelakshmi Sankara Narayanan, Sharmela Sahathevan, Abdul Halim Abdul Gafor, Zulfitri Azuan Mat Daud, Pramod Khosla, Alice Sabatino, Enrico Fiaccadori, Karuthan Chinna, Tilakavati Karupaiah

**Affiliations:** 1 Dietetics Program, Faculty of Health Sciences, Universiti Kebangsaan Malaysia, Kuala Lumpur 50300, Malaysia; khorbanhock@gmail.com (B.-H.K.); sreelakshmiprem07@gmail.com (S.S.N.); sham_0901@yahoo.com (S.S.); 2 Department of Medicine, Faculty of Medicine, Universiti Kebangsaan Malaysia, Kuala Lumpur 56000, Malaysia; halimgafor@gmail.com; 3 Department of Nutrition and Dietetics, Faculty of Medicine and Health Sciences, Universiti Putra Malaysia, Selangor 43400, Malaysia; zulfitri@upm.edu.my; 4 Department of Nutrition & Food Sciences, College of Liberal Arts & Sciences, Wayne State University, Detroit, MI 48202, USA; aa0987@wayne.edu; 5 Acute and Chronic Renal Failure Unit, Department of Clinical and Experimental Medicine, University of Parma, 43126 Parma, Italy; alice.sabatino86@gmail.com (A.S.); enrico.fiaccadori@unipr.it (E.F.); 6 Department of Social and Preventive Medicine, Faculty of Medicine, University of Malaya, Kuala Lumpur 50603, Malaysia; karuthan@gmail.com; 7 School of BioSciences, Faculty of Health and Medical Sciences, Taylor’s University, Subang Jaya 47500, Malaysia

**Keywords:** haemodialysis, nutrition, inflammation, antioxidants, omega-3 fatty acids, polyphenols, fibres, systematic review, meta-analysis

## Abstract

Low-grade chronic inflammation is prevalent in patients undergoing haemodialysis (HD) treatment and is linked to the development of premature atherosclerosis and mortality. The non-pharmacological approach to treat inflammation in HD patients through nutritional intervention is well cited. We aimed to assess the efficacy of different nutritional interventions at improving inflammatory outcomes in HD patients, based on markers such as C-reactive protein (CRP), interleukin-6 (IL-6), and tumour necrosis factor-α (TNF-α). We searched PubMed, Cochrane Library, and Embase for randomized controlled trials (RCT) published before June 2017. Inclusion criteria included RCTs on adult patients on maintenance HD treatment with duration of nutritional interventions for a minimum 4 weeks. Risk of bias was assessed using the Jadad score. In total, 46 RCTs experimenting different nutritional interventions were included in the review and categorized into polyphenols rich foods, omega-3 fatty acids, antioxidants, vitamin D, fibres, and probiotics. Meta-analyses indicated significant reduction in CRP levels by omega-3 fatty acids (Random model effect: −0.667 mg/L, *p* < 0.001) and vitamin E (fixed model effect: −0.257 mg/L, *p* = 0.005). Evidence for other groups of nutritional interventions was inconclusive. In conclusion, our meta-analysis provided evidence that omega-3 fatty acids and vitamin E could improve inflammatory outcomes in HD patients.

## 1. Introduction

Haemodialysis (HD) patients experience a 3 to 4 times a higher mortality rate compared to their peers without chronic kidney disease (CKD). Cardiovascular disease (CVD) is cited as the main cause of mortality [1]. Epidemiological studies show that traditional CVD risk factors such as obesity, hypercholesterolemia, and high blood pressure exhibit paradoxical relationships with mortality risk in this population [2,3]. Chronic low-grade inflammation is a major contributing factor to the pathogenesis of atherosclerosis and has been reported in 30 to 50% of HD patients [4]. Acute inflammation is an adaptive response towards injury and infection, but a dysregulated on-going inflammatory state detrimentally affects the physiological process [5]. Particularly, chronic systemic inflammation in HD patients as indicated by increased levels of inflammatory markers such as C-reactive protein (CRP) and interleukin-6 (IL-6) are strong predictors for CVD and overall mortality [6,7]. Inflammation per se also contributes to the early development of co-morbid conditions such as protein energy wasting, vascular calcification, endocrine disorders, and depression, which vastly decrease the quality of life in HD patients [8,9].

Patients undergoing HD treatment may experience inflammation triggered by both endogenous and exogenous factors. Elevations of CRP and IL-6 levels have been documented in CKD patients even before initiation of HD [10]. The HD procedure itself is directly involved in triggering the inflammatory response by (i) passage of endotoxin from the dialysate to the circulation [9]; (ii) filter membrane bio-incompatibility [11]; (iii) use of a central dialysis catheter [12]; and (iv) non-ultrapure dialysate use [13]. Other contributory factors to inflammation include fluid overload, persistent infections, and periodontal disease [9]. HD patients also experience elevated oxidative stress levels from increased production of reactive oxygen species (ROS) and reduction in antioxidant defences [14]. The relationship between inflammation and oxidative stress is bidirectional and synergic [15]. Oxidative stress invokes inflammation via activation of nuclear factor kappa B (NF-κB), a transcription factor that regulates inflammatory responses with HD patients experiencing higher NF-κB gene expression compared to their healthier counterparts [15,16]. Recently, the gut has been identified as a potential source of inflammation. In CKD patients, the gut microbiome milieu shifts towards favouring the growth of bacteria with urease and co-enzymes generating uremic toxins such as *p*-cresyl and indoxyl sulfates. Translocation of these substances from the gut into the blood compartments induces systemic inflammation as occurs in the case of translocation of living bacteria due to intestinal barrier disruption [17,18].

Since persistent inflammation is a major contributory factor to morbidity and mortality in HD patients, it is identified as a potential target of treatment to improve outcomes. Drugs commonly prescribed to HD patients such as statins, angiotensin-converting-enzyme inhibitor, and angiotensin receptor blockers with pleiotropic anti-inflammatory effects, as well as novel targeted anticytokine treatments, have been suggested as potential therapies to treat inflammation in HD patients [19]. There is also a growing interest in exploring the therapeutic effects of dietary components with immune modulating properties. In fact, modifiable risk factors such as cultural habits and dietary intake may explain discrepancies in CRP levels in HD patients across different nations reported in The Dialysis Outcomes and Practice Patterns Study [6,20]. As the evidence of nutritional interventions in reducing inflammation biomarkers in HD patients is accumulating, we therefore adopted the systematic review approach to identify potential nutritional interventions and examine their efficacy as the anti-inflammatory therapeutic agents for HD patients.

## 2. Materials and Methods

### 2.1. Data Sources, Searches, and Selection

We searched the following database through June 2017: PubMed, Embase, and Cochrane Library to identify all published randomized controlled trials (RCTs) for nutritional intervention in maintenance HD patients that listed inflammatory outcomes. We used “hemodialysis/haemodialysis” AND (“omega-3 fatty acids” OR “alpha-linolenic acids” OR “flaxseed” OR “selenium” OR “vitamin E” OR “tocopherols” OR “tocotrienols” OR “vitamin C” OR “ascorbic acids” OR “antioxidants” OR “alpha-lipoic acids” OR “vitamin D” OR “flavonoids” OR “isoflavones” OR “soy protein” OR “cathechins” OR “green tea” OR “pomegranate” OR “fiber” OR “prebiotics” OR “probiotics”) as the search terms. Citations of studies identified were exported to EndNote version X7.5.3. Two authors (B.H.K. and S.S.N.) independently reviewed the titles and abstracts, and full texts of potential studies were retrieved for further appraisal. In case of disagreement between the two authors, a third author (T.K.) was referred. We also performed a manual search for eligible studies by checking the reference lists of relevant original and review articles.

We included eligible studies meeting these criteria: (i) published randomized controlled (parallel arm and crossover) trials in adult end stage renal disease (ESRD) patients (≥18 years old) undergoing maintenance HD; (ii) comparing nutrition interventions with a placebo or blank control; (iii) at least a 4-week follow up; (iv) reporting at least one of these inflammatory outcomes: either C-reactive protein (CRP) or high sensitivity CRP (hsCRP), interleukin-6 (IL-6), and tumour necrosis factor-α (TNF-α); and (v) English language publications. We excluded (i) studies on paediatric patients, pre-dialysis CKD patients, acute kidney injury patients, ESRD patients with other renal replacement therapy modalities such as peritoneal dialysis and transplant; (ii) studies with provision of nutrition intervention via injection, intravenous route, topical, or dialyzer; (iii) single arm, cross-sectional or prospective studies; and (iv) case reports, conference proceedings, review articles, editorials, and letters.

### 2.2. Quality Assessment

Two authors (B.H.K. and S.S.N.) performed the quality assessment of studies included in this systematic review using the Jadad scale [21]. Based on the study design such as randomization and blinding, as well as withdrawals and dropouts, each study was rated with a score ranging from 0 to 5, and studies with Jadad score above 3 were considered high quality.

### 2.3. Statistical Analysis

A meta-analysis was planned whenever a minimum 3 studies with similar nutrition interventions were available. Studies were excluded from the meta-analysis when data could not be retrieved from the manuscript or when there was a lack of response from primary authors contacted for critical data for such manuscripts. Two meta-analyses were performed to test the effects of omega-3 fatty acids supplementation and vitamin E supplementation on CRP, respectively. Extractable data for each study was tabulated. Standard mean difference (SMD) and 95% confidence intervals (CIs) were calculated from sample size, pre- and post-mean, and standard deviation changes for both intervention and control groups. *I*^2^ statistic was used to assess the heterogeneity between study results in meta-analyses, and *I*^2^ above 75% was considered as high heterogeneity. In the presence of heterogeneity, the random effect model was used to pool data from relevant studies. STATA software (version 12.0, StataCorp, College Station, TX, USA) was used for the analyses.

## 3. Results

**Figure 1:**
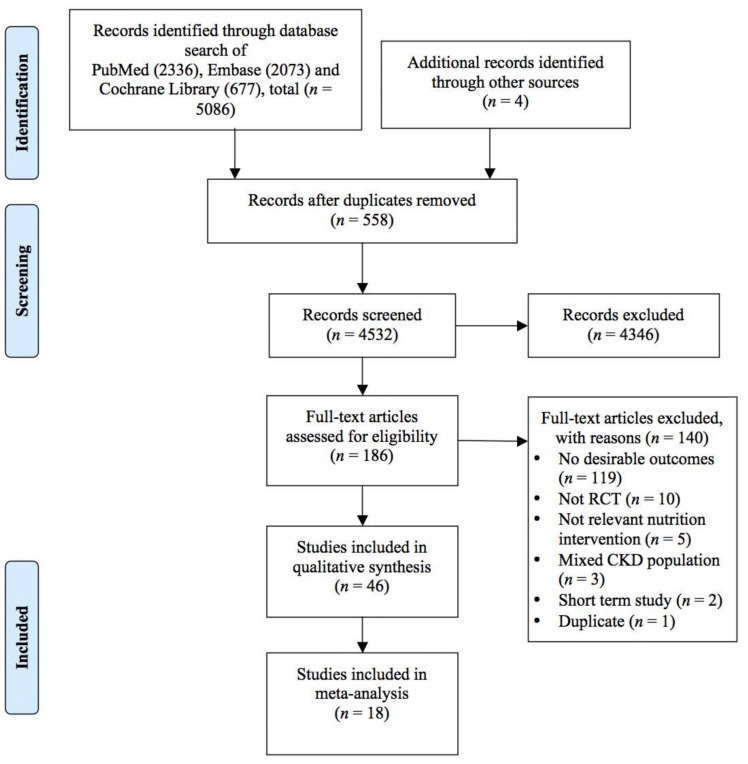
Preferred Reporting Items for Systematic Reviews and Meta-Analyses (PRISMA) study flow for literature search and trials selection process. Abbreviation: RCT, randomized controlled trials; CKD, chronic kidney disease.

Forty-six studies out of 186 studies (Table S1) met the inclusion criteria (Figure 1). Table 1 summarises the characteristics of studies included for this review. The nutrition interventions were categorized into 6 main groups: (i) polyphenols [22,23,24,25,26,27,28,29]; (ii) omega-3 fatty acids [30,31,32,33,34,35,36,37,38,39,40,41,42]; (iii) antioxidants [39,43,44,45,46,47,48,49,50,51,52,53,54,55]; (iv) vitamin D [56,57,58,59]; (v) fibre and probiotics [60,61,62,63]; and (vi) combinations of more than one type of nutrition intervention [39,47,64,65,66,67]. Two studies included had 3 intervention arms [39,47]. Forty-four studies were randomized parallel studies and 2 were randomized crossover studies. The sample size ranged from 10 to 325 patients and only 8 studies had total sample size above 100 patients [25,29,38,40,44,59,62,66]. The study duration ranged from one to 12 months. However, only one study lasted 12 months [25], while others were 6 months or below. Seventeen studies originated from North America, 15 from Middle East, 7 from Europe, 5 from Asia, and 2 from South America. CRP was reported in 44 studies, whereas IL-6 and TNF-α were reported in 19 and 9 studies, respectively. As per the Jadad scoring [21] to rate quality of these studies, one study [23] had a score of 1, 10 studies [24,41,43,44,45,47,52,53,54,62] scored 2, 9 studies [22,33,35,49,55,57,60,61,65] scored 3, 12 studies [25,26,28,29,32,34,36,40,46,50,51,67] scored 4, and 14 studies [27,30,31,37,38,39,42,48,56,58,59,63,64,66] scored 5.

### 3.1. Polyphenols

Polyphenol-rich nutrition interventions (total *n* = 381, studies = 8) used soy isoflavones (total *n* = 69, studies = 3), pomegranate juices (*n* = 101) and extract (*n* = 27), cocoa flavanols (*n* = 52), turmeric powder (*n* = 100), and grape powder (*n* = 32, study = 1). The large heterogeneity of polyphenol therapies did not allow for a formal meta-analysis. Comparing soy isoflavones to either milk or whey protein, all 3 studies reported no significant reduction in CRP levels [22,23,24], while one study reported significant reduction in IL-6 levels [24]. Shema-didi et al. [25] reported significant reduction in IL-6 and TNF-α levels after consumption of pomegranate juice for one year. Contrarily, another study (*n* = 27) reported that pomegranate extract consumption did not lead to significant reduction in both CRP and IL-6 levels [26]. Pokferat et al. [29] reported significant reduction in CRP levels after 8 weeks of turmeric powder supplementation. Two studies with the short intervention period (4–5 weeks) examining the effects of cocoa flavanols [27] or grape powder [28] reported no significant reduction in CRP and IL-6 levels.

### 3.2. Omega-3 Fatty Acids

**Figure 2:**
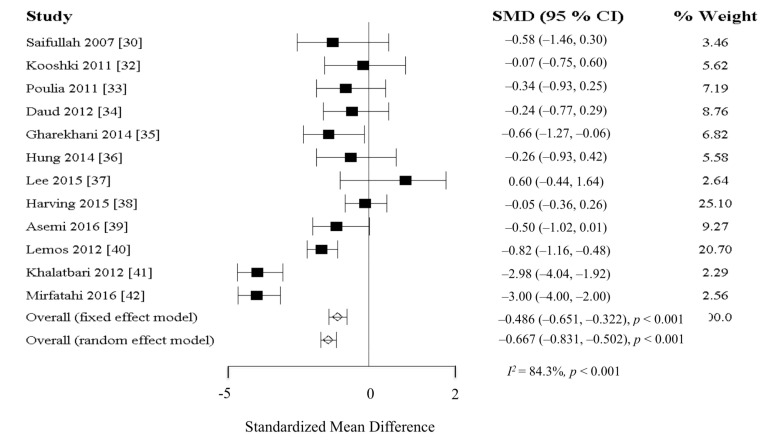
Mean changes in C-reactive protein after omega-3 fatty acids supplementation. Abbreviation: CI, confidence interval; SMD, Standard mean difference.

Thirteen studies investigated the effect of omega-3 fatty acid supplementation on the reduction of inflammation in HD patients. Ten studies (total *n* = 486) used eicosapentaenoic acid (EPA) and docosaahexanoic acid (DHA) from fish oil, while 3 studies (total *n* = 209) used alpha-linolenic acid (ALA) from flaxseed oil. The dosage of EPA and DHA ranged between 600 to 1933 mg/day and 300 to 967 mg/day, respectively. Two studies [30,31] comparing EPA and DHA to corn oil or soybean oil reported significant reduction in CRP levels. Contrarily, 8 studies [32,33,34,35,36,37,38,39] comparing EPA and DHA to olive oil, paraffin oil, or placebo reported no significant reduction in CRP, IL-6, and TNF-α levels. Three studies [40,41,42] using flaxseed or flaxseed oil reported significant reduction in CRP levels. One study [31] was excluded from the meta-analysis due to lack of required data. Meta-analysis including all 12 studies (*n* = 662) reported significant difference in CRP (fixed model: SMD = −0.486 mg/L, 95% CI = −0.831, −0.502, *p* < 0.001; random model: SMD = −0.667 mg/L, 95% CI = −0.651, −0.322; *I*^2^ = 84.3%; *p* < 0.001) (Figure 2). In the subgroup analysis for different forms of omega-3 fatty acids, significant reductions in CRP were observed in studies using EPA and DHA (fixed model: SMD = −0.230 mg/L, 95% CI = −0.395, −0.066, *p* = 0.011; random model: SMD = −0.238 mg/L, 95% CI = −0.402, −0.073, *p* = 0.009; *I*^2^ = 7.0%; *p* = 0.377; *n* = 9 studies with 473 participants) or flaxseed (fixed model: SMD = −1.232 mg/L, 95% CI = −1.397, −1.068, *p* < 0.001; random model: SMD = −2.218 mg/L, 95% CI = −2.383, −2.054, *p* < 0.001; *I*^2^ = 93.4%; *p* < 0.001; *n* = 3 studies with 209 participants).

### 3.3. Antioxidants

**Figure 3:**
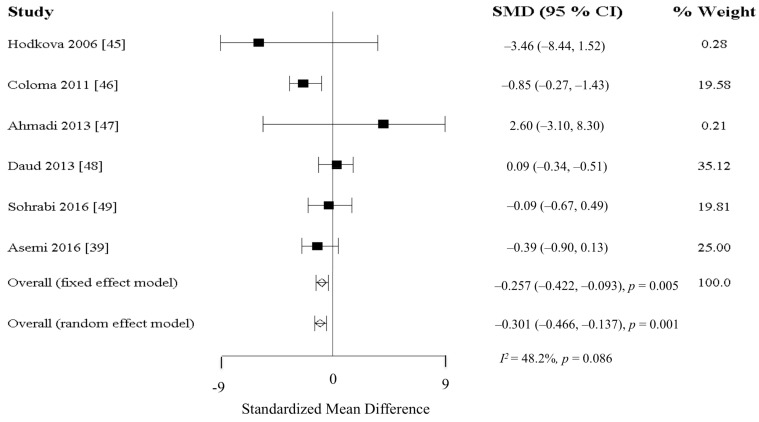
Mean changes in C-reactive protein after vitamin E supplementation. Abbreviation: CI, confidence interval; SMD, Standard mean difference.

There were 14 studies examining the efficacy of antioxidants, including vitamin C, vitamin E, selenium, and α-lipoic acids on inflammatory outcomes. Of these, only the vitamin E studies were sufficient in number to permit a formal meta-analysis. Two studies [43,44] (total *n* = 133) investigated supplementation with oral vitamin C, but only one of these, with a bigger sample size and a higher vitamin C dosage (200 mg/day), achieved significant reduction in CRP levels [44]. Another 6 studies (total *n* = 350) investigated the effects of vitamin E isoforms, tocopherols (TP, *n* = 5) [39,45,46,47,49], and tocotrienols (TT, *n* = 1) [48] on inflammatory markers. All 6 studies reported no significant reduction in CRP levels. Two studies [49] using TP reported significant reduction in IL-6 levels post-intervention, while another study [48] using TT did not observe any significant reduction in IL-6 levels. Meta-analysis of all 6 studies (*n* = 310) reported significant difference in CRP (Fixed model: SMD = −0.257 mg/L, 95% CI = −0.422, −0.093, *p* = 0.005; Random model: SMD = −0.301 mg/L, 95% CI = −0.466, −0.137, *p* = 0.001; *I*^2^ = 48.2%; *p* = 0.086) (Figure 3). The anti-inflammatory effect of selenium was examined in 2 studies [50,51] (total *n* = 124) with similar dose (200 μg) and study duration (3 months). Both studies reported no significant reduction in CRP levels, but significant reduction in IL-6 was reported in one of the studies [50]. Five studies [47,52,53,54,55] (total *n* = 251) examined the effects of α-lipoic acids supplementation with similar dosage at 600 mg/day for 2 to 3 months. Of the 5 studies, only one study [54] reported significant reduction in CRP levels, while non-significant reduction in IL-6 levels [47,53] and TNF-α levels [53,55] were reported.

### 3.4. Vitamin D

Four studies [56,57,58,59] (*n* = 322) examined the effects of different forms of oral nutritional vitamin D on inflammatory biomarkers, and all reported no significant reduction in either CRP, IL-6, or TNF-α levels. Although 4 studies were identified, the lack of required data did not permit a formal meta-analysis.

### 3.5. Fibres and Probiotics

Two studies [60,62] (total *n* = 164) investigated the effects of soluble fibre supplementation in HD patients. One study showed that consumption of 10 g high amylose cornstarch per day did not lead to significant reduction in CRP levels [60]. Contrarily, Xie et al. [62] reported significant reduction in CRP, IL-6, and TNF-α levels by giving either 10 g/day or 20 g/day of water-soluble fibre. One randomized parallel-arm study [63] (*n* = 60) and one randomized crossover study [61] (*n* = 22) compared probiotics of different strains to placebo in lowering CRP levels. CRP was the only inflammatory marker reported in both studies, and only 1 study demonstrated significant reduction in CRP levels [63]. The insufficient number of studies for either fibres or probiotics did not allow for a formal meta-analysis.

### 3.6. Combinations

Seven studies examined the efficacy of nutrient combinations in lowering inflammatory markers. Of these, 2 studies [39,64] (total *n* = 123) used a combination of vitamin E with omega-3 fatty acids. Both reported no significant reduction in CRP levels, while one study [64] reported significant reduction in IL-6 levels. Another 2 studies [47,66] (total *n* = 373) investigated the effects of a combination of vitamin E and α-lipoic acids on CRP and IL-6 levels. Both studies reported no significant reduction in CRP levels. Although one study reported significant reduction in IL-6 levels [47], this effect was not observed in another study with a bigger sample size (*n* = 325) and longer study duration of 6 months [66]. One study [65] (*n* = 37) reported supplementation with an antioxidant combination of vitamin E, vitamin C, and mixed vitamin B for 2 months, but CRP and IL-6 levels did not reduce. Another study [67] (*n* = 42) compared the effects of combination therapy with symbiotic, omega-3 fatty acids, and antioxidants versus placebo for 2 months and concluded that there was no significant reduction in CRP, IL-6, and TNF-α levels.

## 4. Discussion

**Figure 4:**
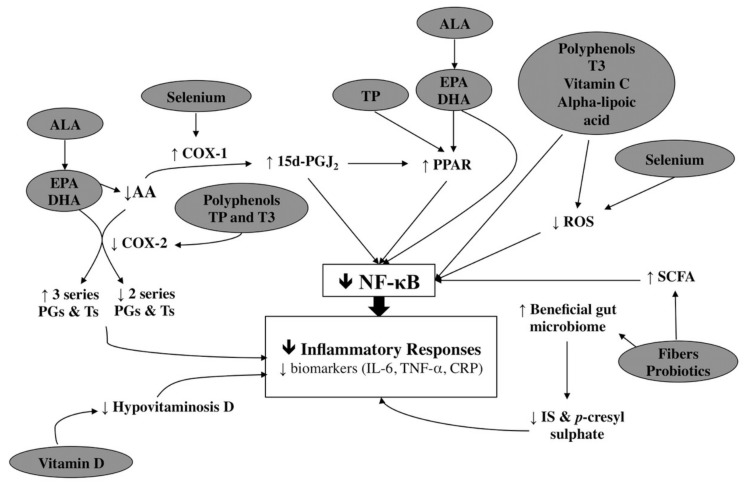
Proposed anti-inflammatory pathways based on reviewed nutritional interventions. Note: NF-κB is involved in the regulation of inflammatory response [16]. Polyphenols [71], omega-3 fatty acids, TP [72], vitamin C [73], alpha-lipoic aicids [74], and selenium [75] inhibit activation of NF-κB by scavenging the ROS. PPAR, a transcription factor that inhibits activation of NF-κB, is upregulated by TP, EPA, and DHA [72,76]. TT suppresses NF-κB gene expression directly [77]. Selenium promotes utilization of AA by COX-1 to generate 15d-PGJ_2_, an anti-inflammatory prostaglandin that inhibits NF-κB [78]. AA is metabolised by COX-2 to produce pro-inflammatory eicosanoids (PGs and Ts). EPA and DHA compete with AA for the substrate in the lipoxygenase and cyclooxygenase pathway to generate less pro-inflammatory substrates [76]. Polyphenols, TP, and TT inhibit the activity of COX-2 [79,80]. Fibres or probiotics stimulate the growth of saccharolytic bacteria and reduce the production of uremic toxins in the gut [18]. In addition, fermentation of soluble fibre produces SCFA, which inhibit the activities of NF-κB [81]. Abbreviations: 15d-PGJ2, 15-deoxy-Δ12,14-prostaglandin J2; AA, arachidonic acid; ALA, alpha-linolenic acid; CRP, C-reactive protein; COX, cyclooxygenase; EPA, eicosapentaenoic acid; DHA, docosahexaenoic acid; IL-6, interleukin 6; IS, indoxyl sulphate; NF-κB, nuclear factor kappa B; PJ, pomegranate juice; PGs, prostaglandins; PPAR, peroxisome proliferators active receptors; ROS, reactive oxygen species; SCFA, short chain fatty acids; TT, tocotrienols; Ts, thromboxanes; TNF-α, tumor necrosis factor alpha; TP, tocopherols. Footnote: ↓, reduce; ↑, increase.

Our systematic review indicates that a large literature exists comprised of 46 RCTs of multiple types of nutritional interventions aimed at moderating inflammatory outcomes in HD patients. Figure 4 provides a schematic representation of potential mechanisms for the wide scope of nutritional interventions as anti-inflammatory agents in HD patients. The meta-analysis from the systematic review provides evidence that omega-3 fatty acids and vitamin E are able to reduce CRP levels in HD patients. For other nutritional intervention studies, the available evidence remains weak and contradictory.

Three previous systematic reviews have evaluated the effects of individual nutritional interventions on inflammatory markers in HD patients. He et al. [68], reviewing 6 studies (*n* = 220), indicated that omega-3 fatty acids in the form of EPA and DHA could reduce CRP levels but not IL-6 and TNF-α. Another review by Xu et al. [69] included 9 studies on omega-3 fatty acids (EPA, DHA, and alpha-linolenic acid (ALA)) in a mixed population of HD, peritoneal dialysis, and non-dialysed stage 5 CKD patients. This review further supported the ability of omega-3 fatty acids to lower CRP levels. Another meta-analysis by Marx et al. [70] with 8 studies focusing on different polyphenol groups concluded that there was a lack of significant improvement in CRP and IL-6 levels in HD patients from the pooled results. Compared to single nutritional intervention approaches, our purpose was to evaluate RCTs inclusive of a wide range of nutritional interventions. As HD patients experience inflammation triggered through multiple sources, evaluating a full spectrum of evidence allowed us to identify potential nutrition intervention beneficial to inflammatory outcomes in HD patients (Figure 4).

### 4.1. Polyphenols

Polyphenols are natural compounds with antioxidant and anti-inflammatory properties, commonly found in edible plant sources. Polyphenols can be distinguished into 4 groups: phenolic acids, flavonoids, stilbenes, and lignans, based on the number of phenol rings that they possess and on the structural elements that bind these rings to one another [82]. The mechanisms for mitigation of inflammatory response using polyphenols have been explored in in vivo and in vitro studies, which include (i) restoring antioxidant activities; (ii) inhibition of the pro-inflammatory enzyme; and (iii) modulation of messengers and transcription factors involved in the process of inflammation [71]. Epidemiological studies have also suggested that consumption of isoflavones may be associated with lower CRP levels in healthy populations [83] and HD patients [84]. However, our systematic review did not generate sufficient evidence to support efficacy of anti-inflammatory properties of polyphenols for HD patients, because reductions in inflammatory markers were not consistently reported in RCTs. Nevertheless, 2 RCTs with bigger sample size (≥100 participants) reported positive outcomes using pomegranate juices [25] and turmeric [29]. The wide variation in polyphenols bioavailability in humans is likely to affect serum response or metabolite generation, which perhaps explains why potential health benefits are not consistently observed in these studies [82].

### 4.2. Omega-3 Fatty Acids

The meta-analysis has provided evidence to support the use of omega-3 fatty acids as anti-inflammatory therapy, either ALA or EPA and DHA. The EPA and DHA from the omega-3 polyunsaturated fatty acids (PUFA) family are well-known nutrients that confer cardiovascular benefits, potentially mediated via modulation of inflammatory response [85]. EPA and DHA have been shown to (i) regulate gene expression associated with inflammatory cytokines production such as NF-κB and PPAR-γ; (ii) lower membrane content of arachidonic acid (AA, *n*-6 PUFA); (iii) inhibit AA metabolism; and (iv) compete with AA as substrates for the key enzymes in cyclooxygenase and lipoxygenase pathway and shift the generation of pro-inflammatory metabolites towards the production of less pro-inflammatory metabolites [76,85]. A fine balance in the dietary intake of *n*-3 PUFA and *n*-6 PUFA appears to be a trigger point for the type of eicosanoids generated. In HD patients with pre-existing CVD, supplementation of 1.7 g *n*-3 PUFA (45% EPA and 37.5% DHA) per day for up to 2 years has yielded significant reduction in myocardial infarction, but not in overall CVD events and mortality [86]. In fact, a one-year prospective cohort study with 216 HD patients observed that fish consumption was associated with improved survival [87]. The ALA also belongs to the omega-3 PUFA family and is an essential nutrient in human diet [88]. Although ALA is a metabolic precursor of EPA and DHA, in vivo conversion of ALA to EPA and DHA in humans is relatively inefficient and large amounts of ALA is required [85]. Therefore, the anti-inflammatory effects of ALA may also be attributed to inhibition of NF-κB and down regulation of the inflammatory gene expression [89]. A meta-analysis by Su et al. [90] concluded that dietary ALA had no effects on blood inflammatory markers in a mixed population. However, the author suggested that anti-inflammatory effects of ALA might only be evident in populations with elevated inflammatory markers. 

### 4.3. Antioxidants

Oxidative stress is closely linked to inflammation; therefore the anti-inflammatory effects of nutrients with antioxidant properties, namely vitamin C, vitamin E, selenium, and alpha-lipoic acids, were identified as typical anti-inflammatory agents for treating HD patients.

Vitamin C, a water-soluble vitamin with antioxidant properties, is commonly depleted in HD patients due to dietary restriction of vitamin C-rich food such as fruits and vegetables and losses during haemodialysis. In a cross-sectional study (*n* = 117), plasma vitamin C levels in HD patients were inversely correlated with hsCRP levels [91]. However, research on the use of vitamin C in HD patients has mainly focused on anaemia management [92], and the efficacy of vitamin C in modulating inflammatory response in HD patients remains largely unexplored.

On the other hand, more studies on vitamin E were available, predominantly in the form of tocopherols (TP) instead of tocotrienols (TT). Although TP and TT share a close structural similarity, TP and TT have distinct biological activities [93]. The anti-inflammatory effect of both TP and TT in reducing serum CRP levels has been documented in a heterogeneous population [94,95]. A meta-analysis also indicated a reduction in oxidative stress and inflammation outcomes with a dialyzer coated with vitamin E compared to a conventional dialyzer [96]. The Secondary Prevention with Antioxidants of Cardiovascular Disease in End Stage Renal Disease reported that the supplementation of 800 IU/day α-TP achieved reductions in composite CVD endpoints and myocardial infarction without affecting total mortality in HD patients with pre-existing CVD [97]. Although our finding is consistent with previous literature that found favourable effects with vitamin E supplementation, The Provision of Antioxidant Therapy in Haemodialysis study was not included in our meta-analysis due to the use of a combination of nutrients [66]. This study examined the efficacy of a combination of mixed TP (666 IU/day) and α-lipoic acid (600 mg/day) in 355 HD patients for a 6-month duration and reported no significant improvement in any of the inflammatory markers after 6 months of antioxidant therapy.

Selenium (Se) is an essential trace element, which acts as an integral structural component of gluthathione peroxidase (GSH-Px), a well-established enzymatic player against oxidative stress through reducing ROS [75]. HD patients generally have lower blood Se concentrations compared to the general population [98], and serum Se concentrations in HD patients were found to be negatively associated with hsCRP levels [99]. Supplementation with Se-enriched yeast increases plasma Se concentrations in HD patients, but plasma GSH-Px concentrations were not impacted. The kidney is suggested as the main site for plasma GSH-Px synthesis, and low plasma GSH-Px levels in CKD patients therefore may not be related to Se deficiency but may rather be due to the failure of the kidneys to synthesize GSH-Px [100,101]. Therefore, the potential beneficial effect of Se supplementation to lower inflammation in HD patients remains unknown.

Alpha-lipoic acid is another naturally occurring antioxidant, which has been suggested to mitigate oxidative stress associated inflammation by scavenging free radicals and inhibiting the pro-inflammatory pathway [74]. Therapeutic applications of alpha-lipoic acid have been commonly reported in many chronic diseases such as obesity, diabetes, and cognitive dysfunctions [102]. However, limited evidence is available to recommend routine supplementation of alpha-lipoic acid in HD patients.

### 4.4. Vitamin D

Vitamin D deficiency is prevalent and has been associated with inflammation in HD patients [103]. Although a 1-year prospective study reported oral vitamin D supplementation attenuated inflammation by reducing CRP levels in HD patients [104], evidence from existing RCTs does not support the role of nutritional vitamin D in modulating inflammation in HD patients.

### 4.5. Fibers and Probiotics

Prospective cohort studies and experimental human feeding interventions in the general population, as well as in CKD patients, have demonstrated that high dietary fibre is associated with lower inflammatory markers [105,106,107]. A recent meta-analysis of intervention studies on a mixed population reported favourable effects of prebiotic and symbiotic supplementations on systemic inflammation [108]. The benefits of dietary fibre on inflammatory outcomes can be attributed to modulation of bowel flora environment and the pleotropic presence of co-nutrients in high fibre foods such as fruits, vegetables, and wholegrain products. HD patients are known to have lower fibre intake, which is reflective of a suboptimal intake of whole grains, fruits, and vegetables for the purpose of potassium restriction [109]. However, there is insufficient evidence demonstrating that either routine consumption of fibres or probiotics can improve gut microbiome in HD patients and subsequently improve inflammatory outcomes in HD patients.

### 4.6. Limitations

Our review has some limitations. Firstly, we included only papers published in the English language. Second, we included studies providing nutritional intervention through the oral route only, and therefore these findings cannot be extrapolated to the intravenous route of nutrient delivery. Third, the heterogeneity of these studies with diverse nutritional interventions did not allow for formal meta-analyses, with the exception of omega-3 fatty acids and vitamin E studies. In addition, adequacy of haemodialysis and dietary intake or adequacy were not controlled or analysed and could have influenced patients’ response. Methods of inflammatory marker analyses were not known or controlled. On this subject we must add that these inflammatory markers are involved in both acute and chronic inflammatory processes. Therefore, events such as infection and acute illness are likely to contribute to significant within-individual variation [110].

## 5. Conclusions

In conclusion, our systematic review and meta-analysis of the existing literature suggest that omega-3 fatty acids and vitamin E alone are effective in lowering CRP levels in supplemented HD patients. However, available evidence for other anti-inflammatory nutritional therapies remains inconclusive and uncertain due to the small number of studies and sample size of the intervention populations. Future studies designed with optimal sample size to provide adequate statistical power and of longer study duration are required and should be extended beyond inflammation to clinical outcomes such as cardiovascular mortality.

## Figures and Tables

**Table 1:** Characteristics of randomized controlled trials categorized by groups.

No.	Author, Year	Country	Intervention	Content	Control	Duration (Month)	*n*	Mean Age (Year)	Gender (M/F)	Dialysis Vintage (Month)	Outcomes	Jadad Score [21]
**Polyphenols**												
1	Fanti et al., 2006 [22]	USA	Soy protein	Isoflavones (26–54 mg)	Milk protein	2	25	61.0	15/10	≥3 months	↔ CRP, ↔ IL-6, ↔TNF-α	3
2	Siefker et al., 2006 [23]	USA	Soy protein	Isoflavones (52 mg)	Whey protein	1	17	50.3	7/10	13.0	↔ CRP, ↔TNF-α	1
3	Tomayko et al., 2014 [24]	USA	Soy isolate	Isoflavones (40 mg)	Whey protein	6 ^a^	27	52.9	17/10	≥3 months	↔ CRP, ↓ IL-6	2
4	Shema-didi et al., 2012 [25]	Israel	Pomegranate juice	Polyphenol (0.7 mmol)	Placebo juice	12	101	65.9	55/46	27.6	↓ IL-6, ↓ TNF-α	4
5	Wu, 2015 [26]	USA	Pomegranate extract	Gallic acid (600–755 mg)	Placebo	6	27	54.3	17/10	75.5	↔ CRP, ↔ IL-6	4
6	Rassaf et al., 2016 [27]	Germany	Cocoa flavanols	Flavanols (900 mg)	Placebo drink	1	52	65.5	38/14	43 ^b^	↔ CRP, ↔ IL-6	5
7	Janiques et al., 2014 [28]	Brazil	Grape jelly + grape powder	Polyphenol (12–500 mg)	Grape jelly	1.15	32	52.9	18/14	111	↔ CRP	4
8	Pakferat et al., 2014 [29]	Iran	Tumeric	Curcumin 66.3 mg	Placebo	8	100	53.3	60/40	5.0	↓ CRP	4
**Omega-3 Fatty Acids**												
9	Saifullah et al., 2007 [30] ^‡^	USA	Omega 3 Fatty Acid	EPA 854 mg, DHA 488 mg	Soybean/corn oil	3	23	57.5	18/5	N/A	↓ CRP	5
10	Bowden et al., 2009 [31]	USA	Omega 3 Fatty Acid	EPA 960 mg, DHA 600 mg	Corn oil	6	33	60.8	19/14	N/A	↓ CRP	5
11	Kooshki et al., 2011 [32] ^‡^	Iran	Omega 3 Fatty acid	EPA 1240 mg, DHA 760 mg	MCT	2.5	34	50.0	21/13	24.5	↔ CRP, ↔ IL-6, ↔ TNF-α	4
12	Poulia et al., 2011 [33] ^‡^	Greece	Omega 3 fatty acid	EPA 1840 mg, DHA 760 mg, α-TP 8 mg	α-TP	1	25	51.0	16/9	112.8	↔ CRP	3
13	Daud et al., 2012 [34] ^‡^	USA	Omega 3 Fatty Acid	EPA 1800 mg, DHA 600 mg	Olive oil	6 ^a^	55	58.5	32/31	41.4	↔ CRP	4
14	Gharekhani et al., 2014 [35] ^‡^	Iran	Omega 3 Fatty acid	EPA 1080 mg, DHA 720 mg	Paraffin oil	4	45	57.0	25/20	65.9	↔ CRP, ↔ IL-6, ↔ TNF-α	3
15	Hung et al., 2014 [36] ^‡^	USA	Omega 3 Fatty acid	EPA 1933 mg, DHA 967 mg	Placebo	3	34	52.0 ^b^	27/7	47 ^b^	↔ CRP, ↔ IL-6, ↔ TNF-α	4
16	Lee, 2015 [37] ^‡^	Korea	Omega 3 Fatty Acid	EPA 1104 mg, DHA 912 mg	Olive oil	3	15	62.	5/10	≥6 months	↔ CRP	5
17	Harving et al., 2015 [38] ^‡^	Denmark	Omega 3 Fatty Acid	EPA 765 mg, DHA 638 mg	Olive oil	3	162	66.7	96/56	45.6	↔ CRP	5
18	Asemi, 2016 [39] ^‡^	Iran	Omega 3 Fatty Acid	EPA 600 mg, DHA 300 mg and 300 mg other omega-3 fatty acids	Placebo	3	60	57.6	40/20	42.0	↔ CRP	5
19	Lemos et al., 2012 [40] ^‡^	Brazil	Flaxseed oil	2 g	Mineral oil	4	145	57.0	85/60	≥3 months	↓ CRP	4
20	Khalatbari Soltani et al., 2012 [41] ^‡^	Iran	Flaxseed	Fat 13.5 g, fiber 6.7 g	Control	2	30	54.3	16/14	32.5	↓ CRP	2
21	Mirfatahi, 2016 [42] ^‡^	Iran	Flaxseed oil	6 g	MCT oil	2	34	54.3	22/12	32.5	↓ CRP	5
**Antioxidants**												
22	Fumeron et al., 2005 [43]	France	Vitamin C	250 mg	Control	2^a^	33	52.0	20/13	71.5	↔ CRP	2
23	Zhang et al., 2013 [44]	China	Vitamin C	200 mg	Control	3	100	64.4	47/53	48 ^b^	↓ CRP	2
24	Hodkova et al., 2006 [45] ^‡^	Czech Republic	α-TP	888 IU	Control	1.15	29	61.6	10/19	32	↔ CRP	2
25	Coloma et al., 2011 [46] ^‡^	Philippine	α-TP	400 IU	Placebo	2	50	59.7	36/14	N/A	↔ CRP	4
26	Ahmadi et al., 2013 [47] ^‡^	Iran	α-TP	400 IU	Placebo	2	41	46.8	20/21	180	↔ CRP, ↓ IL-6	2
27	Daud et al., 2013 [48] ^‡^	USA	TT	180 mg	Placebo	4	81	58.5	43/38	≥3 months	↔ CRP, ↔ IL-6	5
28	Sohrabi et al., 2016 [49] ^‡^	Iran	α-TP	600 IU	Control	2	69	56.3	37/32	N/A	↔ CRP, ↓ IL-6	3
29	Asemi et al., 2016 [39] ^‡^	Iran	α-TP	400 IU	Placebo	3	60	60.6	40/20	41.4	↔ CRP	5
30	Salehi et al., 2013 [50]	Iran	Selenium	200 μg	Placebo	3	80	52.5	36/44	≥3 months	↔ CRP, ↓ IL-6	4
31	Omrani et al., 2015 [51]	Iran	Selenium	200 μg	Placebo	3	64	58.4	30/34	≥6 months	↔ CRP	4
32	Chang et al., 2007 [52]	Korea	α-lipoic acids	600 mg	Control	3	50	64.5	27/23	N/A	↔ CRP	2
33	Ahmadi et al., 2013 [47]	Iran	α-lipoic acids	600 mg	Placebo	2	44	48.9	23/21	18.1	↔ CRP, ↔ IL-6	2
34	El-Nakib et al., 2013 [53]	Egypt	α-lipoic acids	600 mg	Control	3	44	47.7	24/20	92.4	↔ IL-6, ↔ TNF-α	2
35	Khabbazi et al., 2012 [54]	Iran	α-lipoic acids	600 mg	Control	2	52	53.9	34/18	55.3	↓ CRP	2
36	Safa et al., 2014 [55]	Iran	α-lipoic acids	600 mg	Placebo	2	61	57.2	42/19	79.6	↔ TNF-α	3
**Vitamin D**												
37	Marckmann et al., 2012 [56]	Denmark	Cholecalcife-rol	40,000 IU	Placebo	2	27	N/A	N/A	N/A	↔ CRP, ↔ IL-6	5
38	Hung et al., 2013 [57]	USA	Paracalcitol	As per KDOQI guidelines	Cinacalcet	2	10	48.5	6/4	40 ^b^	↔ CRP, ↔ IL-6	3
39	Seibert et al., 2013 [58]	Germany	Cholecalcife-rol	20,000 IU	Placebo	3	33	67.2	18/15	25.2	↔ CRP, ↔ TNF-α	5
40	Miskulin et al., 2016 [59]	USA	Ergocalcife-rol	50,000 IU	Placebo	6	252	61.1	N/A	42 ^b^	↔ CRP	5
**Fibers & Probiotics**												
41	Sirich et al., 2014 [60]	USA	High amylose corn starch	15 g	Waxy corn starch	1.4	40	56.0	24/16	48.0	↔ CRP	3
42	Natarajan et al., 2014 [61]	USA	Probiotics	*S. thermophilus* KB 19, *L. acidophilus* KB 27, and *B. longum* KB 31 (3 × 10^9^ CFU)	Cream of wheat and psyllium husk	2	22	54	6/16	N/A	↔ CRP	3
43	Xie et al., 2015 [62]	China	Water soluble fiber	10 g and 20 g	Placebo starch	1.5	124	52.8	68/56	22.6	↓ CRP, ↓ IL-6, ↓ TNF-α	2
44	Soleimani et al., 2016 [63]	Iran	Probiotics	*L. acidophilus*, *L. casei*, and *B. bifidum* (2 × 10^9^ CFU/g each)	Placebo	3	60	56.7	40/20	42.6	↓ CRP	5
**Nutrient Combinations**												
45	Himmelfarb et al., 2007 [64]	USA	γ–TP + DHA	γ–TP (308 mg) and DHA (800 mg)	High Oleic Sunflower oil	2	63	59.6	40/23	28.1	↔ CRP, ↓ IL-6	5
46	Kamgar et al., 2009 [65]	USA	Antioxidants	Vitamin E (800 IU), vitamin C (250 mg), B6 (100 mg), B12 (250 μg), and folic acid (10 mg)	Placebo	2	37	52.1	22/15	53.6	↔ CRP, ↔ IL-6	3
47	Ahmadi et al, 2013 [47]	Iran	Vitamin E and α-lipoic acids	Vitamin E (400 IU) and α-lipoic acids (600 mg)	Placebo	2	48	51.1	20/28	17.7	↔ CRP, ↓ IL-6	2
48	Himmelfarb et al., 2014 [66]	USA	Mixed TP and α-lipoic acids	Mixed TP (666 IU) and α-lipoic acids (600 mg)	Placebo	6	325	59.0	143/182	51.5	↔ CRP, ↔ IL-6	5
49	Viramontes-Horner et al., 2015 [67]	Mexico	Synbiotic gel, omega-3 fatty acids, and vitamins	*L. acidophilus* NCFM and *Bifidobacterium lactis* Bi-07 (11 × 10^6^ CFU); inulin (2.31 g) EPA and DHA (1.5 g); and vitamins (complex B, folic acid, ascorbic acid, and vitamin E).	Placebo	2	42	39.8	32/10	60.3	↔ CRP, ↔ IL-6, ↔ TNF-α	4
50	Asemi et al., 2016 [39]	Iran	α-TP, EPA, and DHA	α-TP (400 IU), EPA (600 mg), and DHA (300 mg)	Placebo	3	60	57.4	40/20	40.8	↔ CRP	5

^a^ Provided during dialysis day only; ^b^ median; symbols: ↔ non significance (*p* > 0.05); ↓ significant reduction (*p* < 0.05); ^‡^ studies included for meta-analysis; Abbreviations: α-TP, alpha tocopherol; CRP, C-reactive protein; DHA, docosahexaenoic acid; EPA, eicosapentaenoic acid; F, female; IL-6, interleukin-6; KDOQI, Kidney Disease Outcomes Quality Initiative; M, male; MCT, medium chain triglycerides; N/A, not available; TNF-α, tumor necrosis factor alpha; TT, tocotrienols.

## Supplementary Materials

The following are available online at http://www.mdpi.com/2072-6643/10/4/397/s1, Table S1: Study Selection Based on Inclusion Criteria after Reviewing Full Text.
