# Supplementary material for: Efficacy of Nutritional Interventions on Inflammatory Markers in Haemodialysis Patients: A Systematic Review and Limited Meta-Analysis
Source: Nutrients. 2018 Mar 23;10(4):397. doi: 10.3390/nu10040397 (PMC5946182; doi:10.3390/nu10040397)
Supplement: Supplementary file 1 [file nutrients-10-00397-s001.pdf]

Table S1: Study Selection Based on Inclusion Criteria after Reviewing Full Text

First author, year	Journal	Tittle	Include?	Reason of Exclusion
Abdollahzad, 2009	International Journal of Vitamin and Nutrition Research	Effect of vitamin C supplementation on oxidative stress and lipid profiles in hemodialysis patients	No	No measure of desired outcomes
Adamowicz, 2002	Medical Science Monitor	Effect of erythropoietin therapy and selenium supplementation on selected antioxidant parameters in blood of uremic patients on long-term hemodialysis	No	No measure of desired outcomes
Aguilera, 1993	Nephrology Dialysis Transplantation	Effect of vitamin E administration on erythropoietin values and anaemia in hemodialysis patients	No	No measure of desired outcomes
Ahmadi, 2013	Iranian Journal of Kidney Disease	Effect of alpha-lipoic acid and vitamin E supplementation on oxidative stress, inflammation, and malnutrition in hemodialysis patients	Yes	
Akizawa, 2004	Therapeutic Apheresis and Dialysis	Dose-response study of 22-oxacalcitriol in patients with secondary hyperparathyroidism	No	No measure of desired outcomes
An, 2012	Nutrition Research	Omega-3 fatty acid supplementation increases 1,25-dihydroxyvitamin D and fetuin-A levels in dialysis patients	No	Mixed dialysis population
Ando, 1999	Journal of American Society of Nephrology	Eicosapentanoic acid reduces plasma levels of remnant lipoproteins and prevents in vivo peroxidation of LDL in dialysis patients	No	No measure of desired outcomes
Antoniadi, 2005	Renal Failure	Effect of 1-year oral alpha-tocopherol administration on anticardiolipin antibodies in hemodialysis patients	No	No measure of desired outcomes
Antoniadi, 2008	Therapeutic Apheresis and Dialysis	Effect of one-year oral alpha-tocopherol administration on the antioxidant defense system in hemodialysis patients	No	No measure of desired outcomes
Ardalan, 2007	Nephrology Dialysis Transplantation	Vitamin E and selenium co-supplementation attenuates oxidative stress in haemodialysis patients receiving intra-dialysis iron infusion	No	No measure of desired outcomes
Armas, 2012	Clinical Journal of American Society of Nephrology	25-Hydroxyvitamin D response to cholecalciferol supplementation in hemodialysis.	No	No measure of desired outcomes
Asemi, 2016	Molecular Nutrition and	Effect of the omega-3 fatty acid plus vitamin E supplementation on subjective	No	No measure of

	Food Research	global assessment score, glucose metabolism, and lipid concentrations in chronic hemodialysis patients		desired outcomes
Asemi, 2016	International Urology and Nephrology	Effects of omega-3 fatty acid plus alpha-tocopherol supplementation on malnutrition-inflammation score, biomarkers of inflammation and oxidative stress in chronic hemodialysis patients	Yes	
Baldi, 2013	Journal of Nephrology	Effects of hemodialysis and vitamin E supplementation on low-density lipoprotein oxidizability in end-stage renal failure	No	No measure of desired outcomes
Beavers, 2009	Journal of Renal Nutrition	Effect of over-the-counter fish-oil administration on plasma Lp(a) levels in an end-stage renal disease population	No	No measure of desired outcomes
Begum, 2004	Journal of Renal Nutrition	Supplementation with n-3 and n-6 polyunsaturated fatty acids: effects on lipoxygenase activity and clinical symptoms of pruritus in hemodialysis patients	No	No measure of desired outcomes
Bhan, 2015	Clinical Journal of American Society of Nephrology	Nutritional vitamin D supplementation in dialysis: a randomized trial.	No	No measure of desired outcomes
Bhogade, 2008	Indian Journal of Clinical Biochemistry	Effect of vitamin E supplementation on oxidative stress in hemodialysis patients.	No	No control.
Biniaz, 2014	Iranian Journal of Kidney Diseases	Effect of Vitamin C Supplementation on Serum Uric Acid in Patients Undergoing Hemodialysis A Randomized Controlled Trial	No	No measure of desired outcomes
Biniaz, 2015	Saudi Journal if Kidney Diseases and Transplantation	Effect of vitamin C supplementation on marital satisfaction in patients undergoing hemodialysis: A randomized, double-blind and placebo-controlled trial	No	No measure of desired outcomes
Boaz, 2000	Lancet	Secondary prevention with antioxidants of cardiovascular disease in endstage renal disease (SPACE): randomised placebo-controlled trial	No	No measure of desired outcomes
Bonomini, 1995	Nephrology Dialysis Transplantation	Effects of selenium supplementation on immune parameters in chronic uraemic patients on haemodialysis	No	No measure of desired outcomes
Bowden, 2007	Journal of Renal Nutrition	Effects of omega-3 fatty acid supplementation on vascular access thrombosis in polytetrafluorethylene grafts.	No	No measure of desired outcomes
Bowden,	Nutrition in Clinical	Fish oil supplementation lowers C-reactive protein levels independent of	Yes	

2009	Practice	triglyceride reduction in patients with end-stage renal disease		
Calo, 2014	Clinical Nutrition	Molecular biology based assessment of green tea effects on oxidative stress and cardiac remodelling in dialysis patients	No	No measure of desired outcomes
Candan, 2002	Cell Biochemistry and Function	Effect of vitamin C and zinc on osmotic fragility and lipid peroxidation in zinc-deficient haemodialysis patients	No	No measure of desired outcomes
Castilla, 2006	American Journal of Clinical Nutrition	Concentrated red grape juice exerts antioxidant, hypolipidemic, and antiinflammatory effects in both hemodialysis patients and healthy subjects	No	Short term intervention (less than 1 month)
Castilla, 2008	American Journal of Clinical Nutrition	Comparative effects of dietary supplementation with red grape juice and vitamin E on production of superoxide by circulating neutrophil NADPH oxidase in hemodialysis patients	No	Short term intervention (less than 1 month)
Chan, 2006	Nephrology Dialysis Transplantation	Effect of ascorbic acid supplementation on plasma isoprostanes in haemodialysis patients.	No	No measure of desired outcomes
Chang, 2007	American Journal of Nephrology	Effects of alpha-lipoic acid on the plasma levels of asymmetric dimethylarginine in diabetic end-stage renal disease patients on hemodialysis: a pilot study	Yes	
Chao, 2002	The Journal of Nutritional Biochemistry	Vitamin C and E supplements improve the impaired antioxidant status and decrease plasma lipid peroxides in hemodialysis patients small star, filled	No	No measure of desired outcomes
Chen, 2005	American Journal of Kidney Diseases	Variable effects of soy protein on plasma lipids in hyperlipidemic and normolipidemic hemodialysis patients	No	No measure of desired outcomes
Chen, 2006	British Journal of Nutrition	Effect of soya protein on serum lipid profile and lipoprotein concentrations in patients undergoing hypercholesterolaemic haemodialysis	No	No measure of desired outcomes
Coloma, 2011	Philippine Journal of Internal Medicine	Effects of Vitamin E on a Biomarker of Inflammation and Precursors of Atherogenesis in Chronic Hemodialysis Patients	Yes	
Corredor, 2016	Food and Chemical Toxicology	Unfermented grape juice reduce genomic damage on patients undergoing hemodialysis	No	No measure of desired outcomes
Cristol, 1997	Nephrology Dialysis Transplantation	Erythropoietin and oxidative stress in haemodialysis: beneficial effects of vitamin E supplementation	No	No measure of desired outcomes

Cruz-Mora, 2014	Journal of Renal Nutrition	Effects of a symbiotic on gut microbiota in Mexican patients with end-stage renal disease.	No	No measure of desired outcomes
Dashti-Khavidaki, 2014	American Journal of Therapeutics	Effects of Omega-3 Fatty Acids on Depression and Quality of Life in Maintenance Hemodialysis Patients	No	No measure of desired outcomes
Daud, 2012	Vascular Health and Risk Management	Effects of protein and omega-3 supplementation, provided during regular dialysis sessions, on nutritional and inflammatory indices in hemodialysis patients	Yes	
Daud, 2013	Vascular Health and Risk Management	Vitamin E tocotrienol supplementation improves lipid profiles in chronic hemodialysis patients.	Yes	
de Mattos, 2017	Journal of Renal Nutrition	Omega-3 Fatty Acid Supplementation is Associated With Oxidative Stress and Dyslipidemia, but Does not Contribute to Better Lipid and Oxidative Status on Hemodialysis Patients	No	No measure of desired outcomes
Degar, 2016	Clinical Journal of American Society of Nephrology	High Dose Omega-3 Fatty Acid Administration and Skeletal Muscle Protein Turnover in Maintenance Hemodialysis Patients	No	No measure of desired outcomes
Delanaye, 2013	Nephrology Dialysis Transplant	Cholecalciferol in haemodialysis patients: a randomized, double-blind, proof-of-concept and safety study	No	No measure of desired outcomes
Delarue, 2008	British Journal of Nutrition	Fish oil attenuates adrenergic overactivity without altering glucose metabolism during an oral glucose load in haemodialysis patients	No	No measure of desired outcomes
Diepeveen, 2015	Journal of Internal Medicine	Effects of atorvastatin and vitamin E on lipoproteins and oxidative stress in dialysis patients: a randomised-controlled trial	No	No measure of desired outcomes
Donnelly, 1992	Journal of American Society of Nephrology	Effect of n-3 fatty acids from fish oil on hemostasis, blood pressure, and lipid profile of dialysis patients	No	No measure of desired outcomes
El-hennawy, 2010	American Journal of Therapeutics	A Selected Controlled Trial of Supplementary Vitamin E for Treatment of Muscle Cramps in Hemodialysis Patients	No	No measure of desired outcomes
El-Nakib, 2013	International Journal of Nephrology and Renovascular Disease	Role of alpha-lipoic acid in the management of anemia in patients with chronic renal failure undergoing hemodialysis	No	No measure of desired outcomes

Eleftheriadis, 2010	Therapeutic Apheresis and Dialysis	Alpha-tocopherol administration decreases serum urate levels in hemodialysis patients.	No	No measure of desired outcomes
Eljaoudi, 2015	Phytotherapy Research	Consumption of Argan Oil Improves Anti-Oxidant and Lipid Status in Hemodialysis Patients	No	No measure of desired outcomes
Ewers, 2009	Journal of Renal Nutrition	Effects of unsaturated fat dietary supplements on blood lipids, and on markers of malnutrition and inflammation in hemodialysis patients	No	Non-relevant nutrients
Fanti, 2006	Nephrology Dialysis Transplantation	Positive effect of dietary soy in ESRD patients with systemic inflammation--correlation between blood levels of the soy isoflavones and the acute-phase reactants	Yes	
Fijter, 1995	Haematologica	Does Additional Treatment With Fish Oil Mitigate The Side Effects Of Recombinant Human Erythropoietin In Dialysis Patients?	No	No measure of desired outcomes
Fukuda, 2015	PLoS One	Effects of nutritional supplementation on fatigue, and autonomic and immune dysfunction in patients with end-stage renal disease: a randomized, double-blind, placebo-controlled, multicenter trial.	No	Intervention using enteral nutrition supplement
Fumeron, 2005	Nephrology Dialysis Transplantation	Effects of oral vitamin C supplementation on oxidative stress and inflammation status in haemodialysis patients	Yes	
Galli, 2001	Kidney International Supplement	Vitamin E, lipid profile, and peroxidation in hemodialysis patients	No	No measure of desired outcomes
Ghanel, 2012	Iranian Red Crescent Medical Journal	Efficacy of omega-3 fatty acids supplementation in treatment of uremic pruritus in hemodialysis patients: a double-blind randomized controlled trial.	No	No measure of desired outcomes
Gharekhani, 2014	European Journal of Clinical Pharmacology	The effect of omega-3 fatty acids on depressive symptoms and inflammatory markers in maintenance hemodialysis patients: a randomized, placebo-controlled clinical trial.	Yes	
Gharekhani, 2014	Journal of Renal Nutrition	Effects of oral supplementation with omega-3 fatty acids on nutritional state and inflammatory markers in maintenance hemodialysis patients	No	Duplicate of Gharekhani 2014, Eur J Clin Pharmacol
Gharekhani 2016	Iranian Journal of Kidney Disease	Potential Effects of Omega-3 Fatty Acids on Insulin Resistance and Lipid Profile in Maintenance Hemodialysis Patients: a Randomized Placebo-	No	No measure of desired outcomes

		Controlled Trial		
Giray, 2003	Clinica Chimica Acta	The effect of vitamin E supplementation on antioxidant enzyme activities and lipid peroxidation levels in hemodialysis patients	No	No measure of desired outcomes
Guo, 2013	Nutrients	Zinc supplementation alters plasma aluminum and selenium status of patients undergoing dialysis: a pilot study.	No	No measure of desired outcomes
Hansen, 2014	BMC Nephrology	The influence of vitamin D analogs on calcification modulators, N-terminal pro-B-type natriuretic peptide and inflammatory markers in hemodialysis patients: a randomized crossover study	No	No control
Harving, 2015	Clinical Nephrology	n-3 polyunsaturated fatty acids and adiponectin in patients with end-stage renal disease.	Yes	
Himmelfarb, 2007	Journal of Renal Nutrition	Gamma-tocopherol and docosahexaenoic acid decrease inflammation in dialysis patients	Yes	
Himmelfarb, 2014	Journal of American Society of Nephrology	Provision of antioxidant therapy in hemodialysis (PATH): a randomized clinical trial	Yes	
Hodkova, 2005	Renal Failure	Influence of parenteral iron therapy and oral vitamin E supplementation on neutrophil respiratory burst in chronic hemodialysis patients	No	No measure of desired outcomes
Hodkova, 2006	Renal Failure	Influence of oral vitamin E therapy on micro-inflammation and cardiovascular disease markers in chronic hemodialysis patients	Yes	
Hsu, 2007	American Journal of Clinical Nutrition	Chronic green tea extract supplementation reduces hemodialysis-enhanced production of hydrogen peroxide and hypochlorous acid, atherosclerotic factors, and proinflammatory cytokines	No	No randomization
Hung, 2013	Journal of Renal Nutrition	A pilot study of active vitamin D administration and insulin resistance in African American patients undergoing chronic hemodialysis	Yes	
Hung, 2015	Nephrology Dialysis Transplant	Omega-3 fatty acids inhibit the up-regulation of endothelial chemokines in maintenance hemodialysis patients	Yes	
Ibrahim, 2015	Hemodialysis International	Study of the effect of vitamin D supplementation on glycemic control in type 2 diabetic prevalent hemodialysis patients	No	No measure of desired outcomes
Irish, 2017	JAMA Internal Medicine	Effect of Fish Oil Supplementation and Aspirin Use on Arteriovenous Fistula Failure in Patients Requiring Hemodialysis: A Randomized Clinical Trial	No	No measure of desired outcomes

Islam, 2000	Atherosclerosis	Alpha-tocopherol supplementation decreases the oxidative susceptibility of LDL in renal failure patients on dialysis therapy	No	No measure of desired outcomes
Inal, 1999	Free Radical Research	Antioxidant status and lipid peroxidation in hemodialysis patients undergoing erythropoietin and erythropoietin-vitamin E combined therapy	No	No measure of desired outcomes
Jabbari, 2016	Romanian Journal of Internal Medicine	The Effect of Omega-3 Supplement on Serum Lipid Profile in Patients Undergoing Hemodialysis: A Randomized Clinical Trial	No	No measure of desired outcomes
Janiques, 2014	Journal Brasileiro de Nerfrologia	Effects of grape powder supplementation on inflammatory and antioxidant markers in hemodialysis patients: a randomized double-blind study	Yes	
Jean, 2008	Nephrology Dialysis Transplant	Daily oral 25-hydroxycholecalciferol supplementation for vitamin D deficiency in haemodialysis patients: effects on mineral metabolism and bone markers	No	No measure of desired outcomes
Kajbaf, 2016	Journal of Research in Pharmacy Practice	Does Omega-3 supplementation decrease carotid intima-media thickening in hemodialysis patients?	No	No measure of desired outcomes
Kalantar-Zadeh, 2005	Journal of Renal Nutrition	An anti-inflammatory and antioxidant nutritional supplement for hypoalbuminemic hemodialysis patients: a pilot/feasibility study	No	Intervention using enteral nutrition supplement
Kamgar, 2009	Journal of the National Medical Association	Antioxidant therapy does not ameliorate oxidative stress and inflammation in patients with end-stage renal disease	Yes	
Khabbazi, 2012	Journal of Renal Nutrition	Effects of alpha-lipoic acid supplementation on inflammation, oxidative stress, and serum lipid profile levels in patients with end-stage renal disease on hemodialysis	Yes	
Khajehdehi, 2000	Journal of Renal Nutrition	Lipid-lowering effect of polyunsaturated fatty acids in hemodialysis patients.	No	No measure of desired outcomes
Khajehdehi, 2001	Nephrology Dialysis Transplantation	A randomized, double-blind, placebo-controlled trial of supplementary vitamins E, C and their combination for treatment of haemodialysis cramps	No	No measure of desired outcomes
Khalatbari, 2013	Hemodialysis International	Effects of flaxseed consumption on systemic inflammation and serum lipid profile in hemodialysis patients with lipid abnormalities	Yes	
Kidir, 2015	Renal Failure	Effect of cholecalciferol replacement on vascular calcification and left ventricular mass index in dialysis patients	No	No randomization

Kooshki, 2011	Annals of Nutrition and Metabolism	Effects of marine omega-3 fatty acids on serum systemic and vascular inflammation markers and oxidative stress in hemodialysis patients	Yes	
Kooshki, 2011	Renal Failure	Effects of omega-3 fatty acids on serum lipids, lipoprotein (a), and hematologic factors in hemodialysis patients	No	No measure of desired outcomes
Kuragano, 2014	International Journal of Artificial Organs	Effect of protoconized therapy for renal anemia on adverse events of patients with maintenance hemodialysis	No	No measure of desired outcomes
Lee, 2015	Marine Drugs	The effects of omega-3 fatty acid on vitamin D activation in hemodialysis patients: a pilot study	Yes	
Lemos, 2012	Nutrition Research	Flaxseed oil supplementation decreases C-reactive protein levels in chronic hemodialysis patients	Yes	
Li, 2014	PLoS One	Effect of cholecalciferol supplementation on inflammation and cellular alloimmunity in hemodialysis patients: data from a randomized controlled pilot trial	No	No measure of desired outcomes
Lok, 2012	Journal of American Medical Association	Effect of fish oil supplementation on graft patency and cardiovascular events among patients with new synthetic arteriovenous hemodialysis grafts: a randomized controlled trial	No	No measure of desired outcomes
Løssl, 1999	Lipids	The effect of n-3 fatty acids on leukotriene formation from neutrophils in patients on hemodialysis.	No	No measure of desired outcomes
Lu, 2007	American Journal of Kidney Disease	Serum vitamin E and oxidative protein modification in hemodialysis: a randomized clinical trial	No	No measure of desired outcomes
Maccarrone, 1999	Journal of American Society of Nephrology	Activation of 5-lipoxygenase and related cell membrane lipoperoxidation in hemodialysis patients.	No	No measure of desired outcomes
Mafra, 2009	Nephrology Dialysis Transplantation	Alpha-tocopherol supplementation decreases electronegative low-density lipoprotein concentration [LDL(-)] in haemodialysis patients	No	No measure of desired outcomes
Mann, 2016	Nutrients	The VITAH Trial-Vitamin D Supplementation and Cardiac Autonomic Tone in Patients with End-Stage Kidney Disease on Hemodialysis: A Blinded, Randomized Controlled Trial	No	No measure of desired outcomes
Marckmann, 2012	Nephrology Dialysis Transplantation	Randomized controlled trial of cholecalciferol supplementation in chronic kidney disease patients with hypovitaminosis D	Yes	

Massart, 2014	American Journal of Kidney Diseases	Biochemical parameters after cholecalciferol repletion in hemodialysis: results From the VitaDial randomized trial	No	No measure of desired outcomes
Mazani, 2013	Journal of Renal Nutrition	Effects of zinc supplementation on antioxidant status and lipid peroxidation in hemodialysis patients	No	No measure of desired outcomes
Mazzaferro, 2000	Nephrology Dialysis Transplantation	Changes in bone turnover after parathyroidectomy in dialysis patients: role of calcitriol administration	No	No measure of desired outcomes
Maxwell, 1978	Clinical Pharmacology and Therapeutics	Calcitriol in dialysis patients	No	No measure of desired outcomes
Meireles, 2016	Clinical Nutrition	Effect of cholecalciferol on vitamin D-regulatory proteins in monocytes and on inflammatory markers in dialysis patients: A randomized controlled trial	No	Mixed dialysis population
Merino, 2015	Therapeutic Apheresis and Dialysis	Effects of a single, high oral dose of 25-hydroxycholecalciferol on the mineral metabolism markers in hemodialysis patients	No	No measure of desired outcomes
Mieczkowski, 2014	Medical Science Monitor	Long-term cholecalciferol administration in hemodialysis patients: a single-center randomized pilot study	No	No measure of desired outcomes
Mirfatahi, 2016	International Urology and Nephrology	Effect of flaxseed oil on serum systemic and vascular inflammation markers and oxidative stress in hemodialysis patients: a randomized controlled trial	Yes	
Mirfatahi, 2016	Iranian Journal of Kidney Diseases	Effects of Flaxseed Oil on Serum Lipids and Lipoproteins in Hemodialysis Patients: a Randomized Controlled Trial	No	No measure of desired outcomes
Miskulin, 2016	Journal of American Society of Nephrology	Ergocalciferol Supplementation in Hemodialysis Patients With Vitamin D Deficiency: A Randomized Clinical Trial	Yes	
Moeinzadeh, 2016	Iranian Journal of Kidney Diseases	Effects of Omega-3 Fatty Acid Supplementation on Serum Biomarkers, Inflammatory Agents, and Quality of Life of Patients on Hemodialysis	No	No measure of desired outcomes
Naeini, 2017	Journal of Research in Pharmacy Practice	The Effect of Vitamin D Administration on Intracellular Adhesion Molecule-1 and Vascular Cell Adhesion Molecule-1 Levels in Hemodialysis Patients: A Placebo-controlled, Double-blinded Clinical Trial	No	No measure of desired outcomes
Naini, 2015	Journal of Research in Medical Sciences	The effect of Vitamin D administration on treatment of anemia in end-stage renal disease patients with Vitamin D deficiency on hemodialysis: A placebo-controlled, double-blind clinical trial	No	No measure of desired outcomes
Naini, 2016	Journal of Research in	The effect of vitamin D administration on serum leptin and adiponectin levels	No	No measure of

	Medical Sciences	in end-stage renal disease patients on hemodialysis with vitamin D deficiency: A placebo-controlled double-blind clinical trial		desired outcomes
Nakabayashi, 2011	Nephrology Dialysis Transplant	Effects of synbiotic treatment on serum level of p-cresol in haemodialysis patients: a preliminary study	No	No measure of desired outcomes
Natarajan, 2014	BioMed Rsearch International	Randomized controlled trial of strain-specific probiotic formulation (Renadyl) in dialysis patients	Yes	
Navarro-González, 2013	Journal of Clinical Pharmacology	Anti-inflammatory profile of paricalcitol in hemodialysis patients: a prospective, open-label, pilot study	No	No control
Ohkawa, 2004	Atherosclerosis	Pro-oxidative effect of alpha-tocopherol in the oxidation of LDL isolated from co-antioxidant-depleted non-diabetic hemodialysis patients	No	No measure of desired outcomes
Omrani, 2015	Nephro-Urology Monthly	The Effect of Selenium Supplementation on Acute Phase Reactants and Thyroid Function Tests in Hemodialysis Patients	Yes	
Omrani, 2016	Journal of Renal Injury Prevention	Effect of selenium supplementation on lipid profile in hemodialysis patients.	No	No measure of desired outcomes
Pakfetrat, 2015	Journal of Nephrology	Effects of turmeric on uremic pruritus in end stage renal disease patients: a double-blind randomized clinical trial	Yes	
Pakfetrat, 2015	Hemodialysis International	Role of turmeric in oxidative modulation in end-stage renal disease patients.	No	No measure of desired outcomes
Peck, 1996	American Journal of Clinical Nutrition	Effect of three sources of long-chain fatty acids on the plasma fatty acid profile, plasma prostaglandin E2 concentrations, and pruritus symptoms in hemodialysis patients	No	No measure of desired outcomes
Perunicic-Pekovis, 2007	Nephrology	Effect of n-3 fatty acids on nutritional status and inflammatory markers in haemodialysis patients.	No	No control
Poulia, 2011	Journal of Renal Nutrition	Omega-3 fatty acids supplementation does not affect serum lipids in chronic hemodialysis patients	Yes	
Ramos, 2005	Nefrologia	Lipoprotein oxidation profile in end stage renal disease patients. Role of vitamin C supplementation	No	No measure of desired outcomes

Ramos, 2015	Journal of Renal Nutrition	The short-term effects of olive oil and flaxseed oil for the treatment of constipation in hemodialysis patients	No	No measure of desired outcomes
Rasic-Milutinovic, 2007	Renal Failure	Effects of N-3 PUFAs supplementation on insulin resistance and inflammatory biomarkers in hemodialysis patients	No	No control
Rasmussen, 2010	Nutrition Research	The content of docosahexaenoic acid in serum phospholipid is inversely correlated with plasma homocysteine levels in patients with end-stage renal disease	No	No measure of desired outcomes
Rassaf, 2016	Clinical Journal of American Society of Nephrology	Vasculoprotective Effects of Dietary Cocoa Flavanols in Patients on Hemodialysis: A Double-Blind, Randomized, Placebo-Controlled Trial	Yes	
Rattanasompattikul, 2013	Journal of Cachexia, Sarcopenia and Muscle	Anti-Inflammatory and Anti-Oxidative Nutrition in Hypoalbuminemic Dialysis Patients (AIONID) study: results of the pilot-feasibility, double-blind, randomized, placebo-controlled trial	No	Intervention using enteral nutrition supplement
Ristic-Medic, 2014	Scientific World Journal	Effects of dietary milled seed mixture on fatty acid status and inflammatory markers in patients on hemodialysis	No	No control
Rivara, 2015	Journal of Renal Nutrition	A pilot randomized crossover trial assessing the safety and short-term effects of pomegranate supplementation in hemodialysis patients	No	No control
Rodhe, 2013	Journal of Renal Nutrition	The effect of sea buckthorn supplement on oral health, inflammation, and DNA damage in hemodialysis patients: a double-blinded, randomized crossover study	No	Non-relevant nutrients
Roozbeh, 2011	Renal Failure	Comparative effects of silymarin and vitamin E supplementation on oxidative stress markers, and hemoglobin levels among patients on hemodialysis	No	No measure of desired outcomes
Rusu, 2013	International Urology and Nephrology	The influence of vitamin E supplementation on erythropoietin responsiveness in chronic hemodialysis patients with low levels of erythrocyte superoxide dismutase	No	No measure of desired outcomes
Safa, 2014	International Urology and Nephrology	Effects of alpha lipoic acid supplementation on serum levels of IL-8 and TNF-α in patient with ESRD undergoing hemodialysis	Yes	
Sagheb, 2012	Sleep Medicine	Efficacy of vitamins C, E, and their combination for treatment of restless legs	No	No measure of

		syndrome in hemodialysis patients: a randomized, double-blind, placebo-controlled trial.		desired outcomes
Saifullah, 2007	Nephrology Dialysis Transplantation	Oral fish oil supplementation raises blood omega-3 levels and lowers C-reactive protein in haemodialysis patients--a pilot study	Yes	
Salehi, 2013	Nephrology Dialysis Transplantation	Selenium supplementation improves the nutritional status of hemodialysis patients: a randomized, double-blind, placebo-controlled trial	Yes	
Sato, 2003	Clinical Nephrology	Effects of vitamin supplementation on microcirculatory disturbance in hemodialysis patients without peripheral arterial disease	No	No measure of desired outcomes
Schmitz, 2002	Journal of American Society of Nephrology	Prophylaxis of hemodialysis graft thrombosis with fish oil: double-blind, randomized, prospective trial.	No	No measure of desired outcomes
Seibert, 2013	Nephron Clinical Practice	Influence of cholecalciferol supplementation in hemodialysis patients on monocyte subsets: a randomized, double-blind, placebo-controlled clinical trial	Yes	
Shema-Didi, 2012	Free Radical Biology and Medicine	One year of pomegranate juice intake decreases oxidative stress, inflammation, and incidence of infections in hemodialysis patients: A randomized placebo-controlled trial	Yes	
Shema-Didi, 2013	Nutrition Research	Pomegranate juice intake attenuates the increase in oxidative stress induced by intravenous iron during hemodialysis	No	No measure of desired outcomes
Shema-Didi, 2014	Nutrition Journal	Does Pomegranate intake attenuate cardiovascular risk factors in hemodialysis patients?	No	No measure of desired outcomes
Shirazian, 2013	Journal of Renal Nutrition	The effect of ergocalciferol on uremic pruritus severity: a randomized controlled trial	No	No measure of desired outcomes
Siefker, 2006	Journal of Medicinal Food	Safety and antioxidant effects of a modest soy protein intervention in hemodialysis patients	Yes	
Sinsakul, 1984	American Journal of Clinical Nutrition	Lack of effect of vitamin E therapy on the anemia of patients receiving hemodialysis	No	No measure of desired outcomes
Sirich, 2014	Clinical Journal of American Society of Nephrology	Effect of increasing dietary fiber on plasma levels of colon-derived solutes in hemodialysis patients	Yes	

Siroverm 2008	Renal Failure	Beneficial hematologic effects of daily oral ascorbic acid therapy in ESRD patients with anemia and abnormal iron homeostasis: a preliminary study	No	No measure of desired outcomes
Smith, 2003	Lipids	Vitamin E supplementation increases circulating vitamin E metabolites tenfold in end-stage renal disease patients	No	No measure of desired outcomes
S ørensen, 2015	Journal of Renal Nutrition	The Effect of n-3 Fatty Acids on Small Dense Low-Density Lipoproteins in Patients With End-Stage Renal Disease: A Randomized Placebo-Controlled Intervention Study	No	No measure of desired outcomes
Sohrabi, 2016	American Journal of Kidney Diseases	Intradialytic Oral Protein Supplementation and Nutritional and Inflammation Outcomes in Hemodialysis: A Randomized Controlled Trial	Yes	
Soleimani, 2017	Kidney International	Probiotic supplementation in diabetic hemodialysis patients has beneficial metabolic effects.	Yes	
Sultana, 2016	International Urology and Nephrology	Oral vitamin C supplementation reduces erythropoietin requirement in hemodialysis patients with functional iron deficiency	No	No measure of desired outcomes
Svensson, 2006	Clinical Journal of American Society of Nephrology	N-3 fatty acids as secondary prevention against cardiovascular events in patients who undergo chronic hemodialysis: a randomized, placebo-controlled intervention trial	No	No measure of desired outcomes
Svensson, 2007	Journal of Renal Nutrition	The effect of n-3 fatty acids on heart rate variability in patients treated with chronic hemodialysis.	No	No measure of desired outcomes
Svensson, 2008	Nephrology Dialysis Transplantation	The effect of n-3 fatty acids on lipids and lipoproteins in patients treated with chronic haemodialysis: a randomized placebo-controlled intervention study.	No	No measure of desired outcomes
Svensson, 2010	Journal of Nephrology	The effect of n-3 fatty acids on levels of methylarginines in patients with end-stage renal disease.	No	No measure of desired outcomes
Tabibi, 2017	Hemodialysis International	Effects of flaxseed oil on blood hepcidin and hematologic factors in hemodialysis patients	No	No measure of desired outcomes
Taccone-Gallucci, 2006	Kidney International	N-3 PUFAs reduce oxidative stress in ESRD patients on maintenance HD by inhibiting 5-lipoxygenase activity	No	No measure of desired outcomes
Tayebi-Khosroshahi,	Iranian Journal of Kidney Disease	Effect of omega-3 fatty acid on oxidative stress in patients on hemodialysis.	No	No measure of desired outcomes

2010				
Tayebi-Khosroshahi, 2012	Saudi Journal of Kidney Diseases and Transplantation	Effect of treatment with omega-3 fatty acids on C-reactive protein and tumor necrosis factor-alfa in hemodialysis patients	No	No control
Tayebi-Khosroshahi, 2013	Iranian Journal of Kidney Disease	Effect of omega-3 supplementation on serum level of homocysteine in hemodialysis patients	No	No measure of desired outcomes
Tayebi-Khosroshahi, 2013	Nephro-Urology Monthly	Comparison of vitamin e and L-carnitine, separately or in combination in patients with intradialytic complications	No	No measure of desired outcomes
Taziki, 2007	Saudi Journal of Kidney Diseases and Transplantation	The effect of low dose omega-3 on plasma lipids in hemodialysis patients	No	No measure of desired outcomes
Temple, 2000	Journal of Renal Nutrition	Selenate-supplemented nutritional formula increases plasma selenium in hemodialysis patients	No	No measure of desired outcomes
Tomayko, 2015	Journal of Renal Nutrition	Intradialytic protein supplementation reduces inflammation and improves physical function in maintenance hemodialysis patients	Yes	
Tonelli, 2015	BMC Nephrology	Trace element supplementation in hemodialysis patients: a randomized controlled trial	No	No measure of desired outcomes
Tokmak, 2008	Nephrology Dialysis Transplant	High-dose cholecalciferol to correct vitamin D deficiency in haemodialysis patients	No	No measure of desired outcomes
Túri, 1999	Nephrology Dialysis Transplantation	Erythropoietin and oxidative stress in haemodialysis: beneficial effects of vitamin E supplementation.	No	No measure of desired outcomes
VanBeber, 1995	Journal of Renal Nutrition	The effect of dietary omega-3, -6, and -9 fatty acid supplements on serum fatty acid concentrations in renal dialysis patients: Implications for immune response	No	No measure of desired outcomes
Viramontes-Hörner, 2015	Journal of Renal Nutrition	Effect of a symbiotic gel (Lactobacillus acidophilus + Bifidobacterium lactis + inulin) on presence and severity of gastrointestinal symptoms in hemodialysis patients.	Yes	

Wang, 2016	Journal of Clinical Psychopharmacology	Efficacy of High-Dose Supplementation With Oral Vitamin D3 on Depressive Symptoms in Dialysis Patients With Vitamin D3 Insufficiency: A Prospective, Randomized, Double-Blind Study	No	Mixed dialysis population
Wasio, 2008	Nephron Clinical Practice	Oral vitamin C supplementation in hemodialysis patients and its effect on the plasma level of oxidized ascorbic acid and Cu/Zn superoxide dismutase, an oxidative stress marker	No	No measure of desired outcomes
Wasse, 2014	Journal of Vascular Access	Very high-dose cholecalciferol and arteriovenous fistula maturation in ESRD: a randomized, double-blind, placebo-controlled pilot study	No	No measure of desired outcomes
Weissinger, 2006	Proteomics	Effects of oral vitamin C supplementation in hemodialysis patients: a proteomic assessment.	No	No measure of desired outcomes
Wu, 2015	Journal of Medicinal Food	Effects of Pomegranate Extract Supplementation on Cardiovascular Risk Factors and Physical Function in Hemodialysis Patients	Yes	
Xie, 2015	International Journal of Clinical and Experimental Medicine	Effects of fermentable dietary fiber supplementation on oxidative and inflammatory status in hemodialysis patients	Yes	
Yeksan, 1992	International Journal of Artificial Organs	Effect of vitamin E therapy on sexual functions of uremic patients in hemodialysis	No	No measure of desired outcomes
Yukawa, 1992	Journal of Nutritional Science and Vitaminology	Prevention of aortic calcification in patients on hemodialysis by long-term administration of vitamin E	No	No measure of desired outcomes
Zachara, 2001	Journal of Trace Elements in Medicine and Biology	Selenium and glutathione levels, and glutathione peroxidase activities in blood components of uremic patients on hemodialysis supplemented with selenium and treated with erythropoietin	No	No measure of desired outcomes
Zachara, 2009	Acta Biochimica Polonica	Selenium supplementation to chronic kidney disease patients on hemodialysis does not induce the synthesis of plasma glutathione peroxidase	No	No measure of desired outcomes
Zachara, 2011	Biological Trace Element Research	The effect of selenium supplementation in the prevention of DNA damage in white blood cells of hemodialyzed patients: a pilot study	No	No measure of desired outcomes
Zhang, 2013	BMC Nephrology	Cross-over study of influence of oral vitamin C supplementation on inflammatory status in maintenance hemodialysis patients	Yes	

Zheng, 2016	Nutrients	Cholecalciferol Additively Reduces Serum Parathyroid Hormone and Increases Vitamin D and Cathelicidin Levels in Paricalcitol-Treated Secondary Hyperparathyroid Hemodialysis Patients	No	No measure of desired outcomes
